# Influence of first colostrum pasteurization on serum immunoglobulin G, iron, and activity of gamma-glutamyltransferase in newborn dairy calves

**DOI:** 10.14202/vetworld.2021.2267-2272

**Published:** 2021-08-29

**Authors:** Sebastian Ganz, Klaus Failing, Abdulwahed Ahmed Hassan, Michael Bülte, Axel Wehrend

**Affiliations:** 1 Clinic of Obstetrics, Gynecology and Andrology of Large and Small Animals with Ambulatory Service, Faculty of Veterinary Medicine, Justus-Liebig-University, 35392 Giessen, Hessen, Germany; 2 Biomathematics and Data Processing, Justus-Liebig-University Giessen, 35392 Giessen, Hessen, Germany; 3 Institutes of Veterinary Food Science, Justus-Liebig-University Giessen, 35392 Giessen, Hessen, Germany

**Keywords:** calf, first colostrum, heat treatment, immunoglobulin G, iron deficiency anemia

## Abstract

**Background and Aim:**

Colostrum pasteurization is an established procedure in dairy farms in developed countries. This practice can improve the health status of the offspring by reducing several pathogens. This study aimed to focus on the pasteurization of bovine first colostrum and its influence on certain important bioactive components.

**Materials and Methods:**

This study was conducted in Holstein-Friesian bull calves, which were randomly divided into two groups and fed with 6 L of untreated (UT, n=10) or 6 L of heat-treated (HT, 63.5°C for 30 min, n=10) colostrum from their own dam within the first 12 h after birth. Blood samples were taken before, 24 h, and 48 h after first colostrum intake to determine the concentrations of immunoglobulin G (IgG) and iron and the activity of gamma-glutamyltransferase (GGT) in the serum.

**Results:**

The level of IgG was not affected by pasteurization (p=0.19). However, a slower increase in GGT activity (p<0.05) and a lower serum iron concentration (p=0.04) were observed in the HT group.

**Conclusion:**

It can be concluded that pasteurization influences the absorption of colostrum components and therefore, the passive transfer of immunity, although the level of IgG was not affected by pasteurization in this study.

## Introduction

Bovine colostrum plays an integral role in many aspects of calf development. In the 1^st^ week of life, it is the only source of nutrition for bovine neonates and supplies essential, immunologically active substances. The transfer of various substances present in colostrum induces both passive and active immunization in calves and protects the neonates against infection from pathogens in the environment. Pasteurization of bovine colostrum is an established method for the reduction of microorganisms [1]. Studies have shown that important pathogens such as *Escherichia coli* or *Salmonella* spp. can be inactivated by different temperature-time intervals [2,3]. Furthermore, the concentration of heat-stable pathogens such as *Mycobacterium avium* ssp. *paratuberculosis* (MAP) can be reduced by heat treatment of colostrum, making pasteurization an important technique in hygiene management or for the sanitation of infected farms [4,5]. The temperature-time protocol and the volume of the pasteurized samples are key factors regulating the influence of pasteurization on the concentration of immunoglobulins (Ig) in colostrum and therefore in the serum of neonatal calves [6,7].

Most previous studies were conducted on pooled first colostrum samples that had previously undergone treatment (e.g., deep freezing) before feeding or on samples obtained from the second milking onward [7-9]. This approach neglects the possibility that some components of colostrum may be absorbed differently when maternal colostrum is fed to the own or to a foreign calf; this has already been proven to be true for the absorption of colostral leukocytes [10]; if a calf is fed colostrum from a cow that is not its own mother, the neonate does not absorb the colostral leukocytes [10,11]. To avoid this potential factor, pooling was deliberately omitted in the present study. The first colostrum is different from subsequent milkings because of its composition [12]. Thus, findings from the previous studies cannot be extended to untreated (UT) maternal first colostrum uncritically.

Several studies have examined the influence of pasteurization on bovine colostrum and its impact on the health status of the bovine neonate. However, none of these studies investigated native maternal first colostrum that was fed to individual calves. In a previous study, we developed a temperature-time protocol, which only had a mild influence on the concentration of IgG in bovine first colostrum and its viscosity [13]. We hypothesized that pasteurization of colostrum using the new protocol would have no influence on the IgG levels in calf blood, because in a previous study, we were able to show that vital parameters such as rectal body temperature and respiratory rate were not affected in calves fed with heat-treated (HT) colostrum [14].

The influence of pasteurization on other colostrum components, such as IgG, remains largely unknown. Therefore, in addition to IgG, we selected two important colostrum components as the focus of this study. We investigated the blood iron concentration because iron deficiency anemia is a common disease in neonates, with iron being a key factor in erythropoiesis and synthesis of myoglobin [15]. In general, the iron content in colostrum is low [16,17].

We were interested in the activity of gamma-glutamyltransferase (GGT) in the blood because it is used as an indirect parameter to verify the adequacy of IgG concentration in the postnatal period of calves [18]. The GGT has 1000-times higher activity in colostrum than in mature milk [19]. GGT is absorbed from the colostrum into the neonate and its activity in the blood of the calf can be measured as a marker of colostrum supply. The advantage of this laboratory parameter to detect the failure of passive transfer is that the activity of GGT is not influenced by any disease in the neonate, as is the case when measuring total protein concentration in the serum [20].

The study aimed to investigate the changes in IgG, iron, and GGT concentration in the serum of bovine neonates after feeding with HT first colostrum from the respective dam, without any other processing of the colostrum before feeding.

## Materials and Methods

### Ethical approval

The study was approved by the Regierungspräsidium Gießen (TV G45/2017).

### Background

The study was part of a project investigating the inactivation of MAP in colostrum under different temperature-time combinations of pasteurization. During the course of this project, preliminary tests showed that pasteurization at 63.5°C for 30 min was most effective at reducing MAP, only moderately changing the viscosity of bovine first colostrum [13]. This study was conducted to verify if this procedure can extend for use in neonatal calf feeding.

### Study period and location

The study was conducted from October 2017 to November 2018. The calves were housed in Clinic of Obstetrics, Gynecology and Andrology of Large and Small Animals with Ambulatory Service, Faculty of Veterinary Medicine, Justus-Liebig-University.

### Materials

Twenty Holstein-Friesian bull calves were randomly divided into two groups of ten animals by lot. All calves were born normally, without any assistance from the farmer or a veterinarian. Calves with a birth weight lower than 35 kg were excluded from the study. The calves were separated from their dam immediately after birth and kept in adjacent calf pens. Six liters of first colostrum were milked from every dam and examined with respect to the Ig concentration and the specific gravity (Colostrometer Pfizer, Karlsruhe, Germany). Ten calves (group: UT) received 6 L of UT colostrum from the respective mother cow within the first 6 h of life. The other ten calves (group: HT) were fed 6 L of maternal colostrum, previously heated to 63.5°C for 30 min in a pasteurizer (HT250, Förster, Engen, Germany) directly after collection. All calves were bottle-fed. Calves that consumed <1.5 L of first colostrum within the first 2 h of life were excluded from the study. After an initial intake of 1.5 L within the first 2 h, calves were fed 2.25 L of colostrum every 2 h. Thus, all calves consumed 6 L of maternal colostrum within the first 6 h of life. After a feeding of 6 L of first colostrum, the calves were fed 2 L of a milk replacer (Sanolac Startino®, Sano, Moderne Tierernährung GmbH, Loichingen, Germany) 3 times a day. Between feedings, the calves had access to freshwater from buckets. General clinical examination was performed daily for 10 days. In addition, the navel and joints were examined. All vital parameters were assessed using a score sheet designed by the institution of animal welfare of the Justus-Liebig-University.

### Procedures

Three jugular blood samples (9 mL blood for each collection) were obtained from the calves for laboratory diagnostic analysis. The first blood sample was obtained before colostrum intake within the 1^st^ h of life, and 24 and 48 h after the initial intake of colostrum. The blood samples were sent to the laboratory of the Clinic for Obstetrics, Gynaecology and Andrology of Large and Small Animals without further processing to determine the GGT activity and iron concentration using the LT-SYS® Gamma-GT test kit (Eberhard Lehman GmbH, Germany) and by flame photometry using Efox 5053 from Eppendorf, respectively. The IgG concentration was assayed by a sandwich ELISA in the laboratory at Ludwig-Maximilian-University in Munich [21]. Laboratory methods were used according to Sutter *et al*. [22].

### Statistical analysis

For the statistical estimation of the required sample number, the following assumptions were made: a=0.05; desired power p=0.9 (corresponding to the second type of error probability of β=0.10); smallest medically relevant difference between the groups: ∆ 1.4 standard deviation in the log-normal scale. Under these assumptions, a necessary sample size of n=10 calves was calculated.

The arithmetic means, standard deviations, minima, and maxima were calculated from parameters with an approximately normal distribution. If a right skewed distribution occurred, logarithmic transformation of the data was performed and data description was performed using geometric means and scatter factors. Group comparison was performed as a two-factorial analysis of variance with repeated measurements in the factor time.

A linear mixed model analysis including repeated measurements with respect to time was conducted to determine the impact of the fixed effects group and time as well as the interaction between the two fixed effects. As a random effect, the animal nested in each group was added to the model. The effective size (η) and coefficient-interval (CI) were added to the p-values.

Statistical analyses were performed using the statistics program package SAS 9.4 (SAS® Institute Inc., Cary, NC, USA, 2013).

## Results

### Colostrum quality

The specific gravity of colostrum was 1054±10.13 g/mL in UT and 1057.6±10.2 g/mL in HT.

### IgG

**Figure-1:**
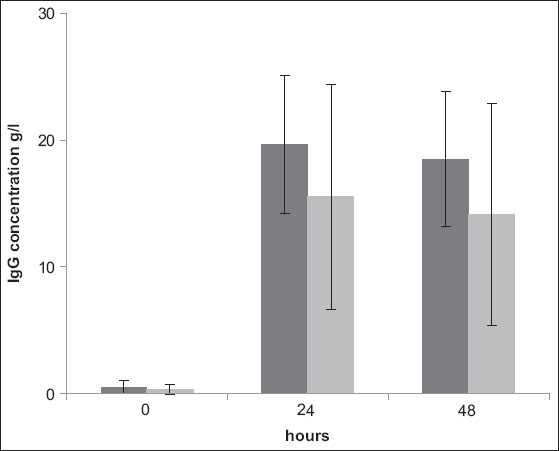
Arithmetic mean and standard deviation of serum IgG concentration in calves in the UT (dark, n=10) and HT (bright, n=10) groups prior to and 24 and 48 h after colostrum intake (p=0.19) UT=Untreated; HT=Heat-treated; GGT=Gamma-glutamyltransferase.

Feeding with pasteurized first colostrum did not affect the IgG concentration in the serum of the calves (p=0.19; η^2^=0.0216-95% CI=[0; 0.138]). The calves in HT had an average IgG concentration of 15.52±5.54 mg/mL compared to 19.63±8.87 mg/mL in UT, 24 h after colostrum intake. The values were 14.12±5.33 mg/mL and 18.47±8.87 mg/mL, respectively, 48 h after feeding (Figure-1).

### Iron

**Figure-2:**
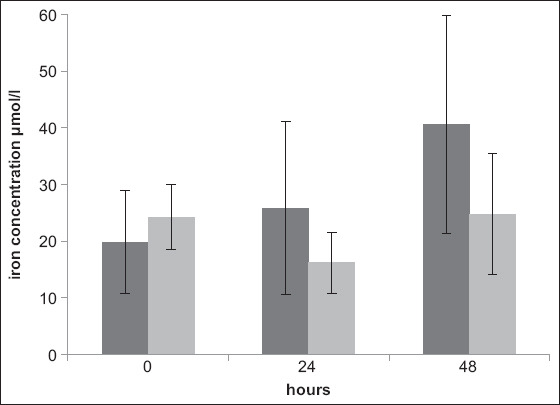
Arithmetic mean and standard deviation of serum iron levels in calves in the UT (dark, n=10) and HT (bright, n=10) groups before and 24 and 48 h after colostrum intake (p=0.04) UT=Untreated; HT=Heat-treated; GGT=Gamma-glutamyltransferase.

The calves in HT showed lower serum iron concentrations (p=0.04; η^2^=0.0657-95% CI=[0; 0.211]) (Figure-2). This influence could also be observed over time between the groups (p=0.005; η^2^=0.1495-95% CI=[0.01; 0.3003]). Nevertheless, the iron concentrations increased over time in both groups. The mean concentrations in UT were 19.86±9.04 μmol/L, 25.79±15.26 μmol/L, and 40.63±19.22 μmol/L (before, 24 h and 48 h after colostrum intake), respectively. In HT, we measured mean values of 24.25±5.70 μmol/L, 16.18±5.46 μmol/L, and 24.79±10.70 μmol/L (before, 24 h and 48 h after colostrum intake), respectively.

### GGT activity

**Figure-3:**
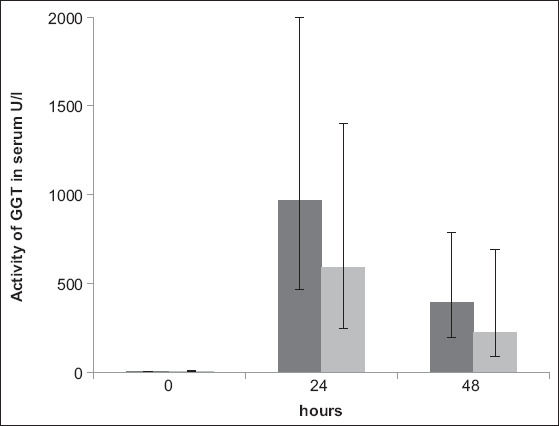
Geometric mean and scattering factor of GGT activity in calves in the UT (dark, n=10) and HT (bright, n=10) groups before and 24 and 48 h after colostrum intake in U/l (p<0.05) UT=Untreated; HT=Heat-treated; GGT=Gamma-glutamyltransferase.

Regardless of the group, all calves exhibited maximum serum GGT activity at 24 h after colostrum intake. Values between 158 U/L and 2750 U/L were measured during this time. Feeding pasteurized colostrum did not have an effect on the activity of GGT at the group level (p=0.3; η^2^=0.0034-95% CI=[0; 0.085]). However, a lower increase was observed among calves in HT during the observation period (p<0.05; η^2^=0.0059-95% CI=[0; 0.0624]) (Figure-3).

## Discussion

Pasteurization of colostrum is an effective method to reduce bacterial contamination [23]. However, the influence of heat treatment on the absorption of colostrum components should be investigated before this method can be recommended without reservation. The previous studies only used pooled or non-individual maternal first colostrum. Thus, their results cannot be uncritically extended to conditions with individual first colostrum. The possibility that the resorption of individual maternal colostrum is different from that of pooled colostrum cannot be excluded [12]. It was proven that colostral leukocytes are only transferred into the neonate if the colostrum is from its own dam [10,11,24].

Several studies have previously investigated the absorption of IgG after intake of HT colostrum; however, the results are contradictory. Our study results demonstrated no effect of the intake of pasteurized colostrum on IgG concentration. Other studies using a temperature-time protocol of 60°C for 60 min have also shown no effect of heat treatment [25,26]. However, other studies detected an elevated IgG concentration in the serum of calves fed with HT colostrum [6,27]. In contrast, there is one study that reports a negative influence on the Ig status after feeding pasteurized colostrum treated at 63°C [7]. Other studies showed that pasteurizing colostrum at 63°C for 30 min causes only a mild or no decrease in IgG concentration in the blood of calves [28,29]. These conflicting results can be explained by the fact that adequate absorption of IgG depends on several factors. Many studies have been conducted on pooled colostrum [6-8]. Pooling of colostrum ensures a uniform concentration of IgG; however, these studies did not consider the individual differences of the first colostrum between cows. In addition, colostral leukocytes are absorbed into the neonatal circulation only after feeding individual maternal colostrum, because only maternal colostral leukocytes can express surface proteins for cellular migration [10,11]. Furthermore, the fed volume [7] and the time of first feeding [30,31] have a decisive influence on an adequate concentration of IgG. These aspects show that sufficient bovine neonatal IgG depends on many factors, and thus, when comparing the results of different studies, attention should be paid to the precise design of the studies.

The concentration of iron in fresh bovine colostrum is approximately 5 mg/kg, compared to 0.0005 mg/kg in mature milk [17,32]. We demonstrated that feeding with pasteurized first colostrum led to a reduction in blood iron concentration without causing any clinical anomalies. A human study reported that the administration of pasteurized colostrum led to a reduced serum iron concentration in babies [33]. This reduction could be attributed to an effect on iron-binding proteins in the colostrum [33]. Due to increased erythropoiesis and postnatal muscle growth, bovine neonates often suffer from iron deficiency anemia due to insufficient iron reserves [15,34]. Therefore, future studies should analyze if feeding with HT colostrum is a risk factor for the development of iron deficiency anemia.

The present study did not show an effect of pasteurization on the activity of GGT in the serum of calves. Therefore, this parameter could be used to control sufficient colostrum intake in the blood of newborn calves after feeding with HT colostrum. However, further studies are required to establish adjusted reference points, as the increase in serum GGT activity was delayed compared to that in calves fed with native colostrum. This observation can be explained by the heat stability of different proteins. In general, the heating of colostrum results in a change in the solubility of almost all whey proteins, an effect that can be observed at temperatures as low as 40°C [35,36]. Gradual denaturation of proteins occurs at temperatures of 60°C and higher due to the destruction of the secondary or tertiary structure caused by the dissolution of non-covalent bonds [37]. In comparison, casein proteins are more resistant to thermal processing, as they contain a higher proportion of stable covalent bonds within the primary structure and can withstand temperatures above 100°C [38,39]. In addition, GGT has higher heat stability compared to other enzymes such as alkaline phosphatase and is completely inactivated only at a temperature of 80°C [40].

## Conclusion

Our study demonstrated that a pasteurization program of 63.5°C for 30 min did not result in an undersupply of IgG to calves. However, the increase in GGT activity after colostrum intake was flattened and the absorption of iron was reduced; therefore, further studies are needed to investigate the extent of adjustment of the current cutoff points for GGT activity after pasteurization, to use this parameter to assess the IgG concentration. In addition, studies are needed to investigate in detail whether the reduction in serum iron concentration after the intake of pasteurized colostrum is a risk factor for the development of iron deficiency anemia.

## Authors’ Contributions

AW: Conceived and supervised the study and had substantial inputs for the analysis and writing of the drafts. SG: Conducted the study and created a first draft of the paper. MB: Co-supervised the study and had substantial inputs for the survey instrument and drafts of the paper. AAH: Performed several experiments on which the present study was based. KF: Conducted the statistical analyses. All authors read and approved the final manuscript.
